# Long non-coding RNA SPRY4-IT1 promotes proliferation and metastasis in nasopharyngeal carcinoma cell

**DOI:** 10.7717/peerj.13221

**Published:** 2022-03-30

**Authors:** Yanfei Li, Zhenpeng Liao, Rong Wang, Zibin Liang, Zhihe Lin, Shiqi Deng, Lei Chen, Zhigang Liu, Shaoyan Feng

**Affiliations:** 1 Department of Otorhinolaryngology, Head and Neck Surgery, The Fifth Affiliated Hospital of Sun Yat-sen University, Zhuhai, Guangdong, China; 2 Guangdong Provincial Key Laboratory of Biomedical Imaging, The Fifth Affiliated Hospital of Sun Yat-sen University, Zhuhai, Guangdong, China; 3 The Cancer Center, The Fifth Affiliated Hospital of Sun Yat-sen University, Zhuhai, Guangdong, China; 4 Department of Neurosurgery, The Fifth Affiliated Hospital of Sun Yat-sen University, Zhuhai, Guangdong, China

**Keywords:** Nasopharyngeal carcinoma, LncRNA SPRY4-IT1, Proliferation, Metastasis

## Abstract

**Background:**

Long non-coding RNA SPRY4 intronic transcript 1 (Lnc RNA SPRY4-IT1) was aberrant-expressed in various kinds of cancer. Increasing evidence demonstrated that lnc RNAs involved in tumorigenesis and metastasis. In this study, we aimed to explore the biological role of SPRY4-IT1 on the phenotype of nasopharyngeal carcinoma (NPC) in vitro and in vivo.

**Methods:**

The expression level of SPRY4-IT1 in NPC cell lines were measured by quantitative real-time polymerase chain reaction (qRT-PCR). Cell Counting Kit-8 (CCK-8) and colony formation assay were used to detect cell proliferation. Wound-healing assay, transwell assay and animal experiment were performed to evaluate the ability of cell migration and metastasis. Cell cycle distribution and apoptosis were determined by flow cytometry. Western blotting and immunofluorescence were employed to identify protein expression.

**Results:**

SPRY4-IT1 was significantly up-regulated in several NPC cell lines (6-10B, CNE-2, and HONE-1) compared with human immortalized nasopharyngeal epithelial cell (NP69). Silencing of SPRY4-IT1 inhibited proliferation, migration, and metastasis, and induced significant G2/M phase arrest and apoptosis. Western blotting showed that the expression levels of cell cycle-related proteins (cyclin B1, cdc2 and p-cdc2) were down-regulated and apoptosis-associated proteins (PARP, cleaved PARP and cleaved caspase-3) were up-regulated after knockdown of SPRY4-IT1. The expression level of E-cadherin was increased and the expression of Vimentin, Snail and Twist1 were decreased after the SPRY4-IT1 knockdown.

**Conclusion:**

lncRNA SPRY4-IT1 played a significant role in NPC proliferation, migration and metastasis, suggesting that SPRY4-IT1 might be a potential therapeutic target for the treatment of NPC.

## Introduction

Nasopharyngeal carcinoma (NPC) is an aggressive malignancy in the head and neck region that usually arises from the epithelium of the nasopharynx. It is generally accepted that NPC is related to multiple etiological factors, such as Epstein-Barr virus (EBV) infection, genetic susceptibility and environmental conditions (Chang et al., 2021; Chen et al., 2019). According to the survey of The International Cancer Research Agency, there were approximately 133,354 cases of newly diagnosed NPC, accounting for 0.7% of all cancers; and approximately 80,008 new deaths, accounting for 0.8% of all cancer deaths around the world in 2020 (Sung et al., 2021). It is striking that more than 70% of NPC patients are first diagnosed with NPC at advanced stages (clinical stages III and IV) owing to the lack of early typical symptoms and its aggressive nature (Siak et al., 2021). Currently, radiotherapy is the mainstay of treatment for NPC patients because of the special anatomical location of the nasopharynx and sensitivity to radiation. With the advances in radiotherapy and chemotherapy, the survival rate of NPC patients has been greatly improved. However, the local recurrence and distant metastasis of NPC patients are the main reason for treatment failure (Su, She & Shen, 2020; Wei et al., 2019; Wong et al., 2021). Therefore, it is significant to further explore deep molecular mechanisms of NPC progression and metastasis, and find new potential therapeutic targets for NPC.

According to the study of the human genome sequence, we know that more than 90% of the genome is transcribed. However, a large proportion of transcripts are non-protein-coding RNAs (International Human Genome Sequencing C, 2004). LncRNAs are the subgroup of the non-protein-coding RNAs (length >200 nucleotides), and were divided into the following categories: intergenic, intronic, enhancer, sense, antisense, and bidirectional base on genomic location (Mattick & Rinn, 2015; Teng et al., 2021). Accumulating studies showed that lncRNAs play an important role in the regulation of biological processes, such as modulating the activity or localization of proteins, serving as organizational frameworks of subcellular structures, and participating in the processing of other RNAs (Wilusz, Sunwoo & Spector, 2009). Of note, the dysregulation of lncRNAs was associated with the tumor formation and progression in many human malignancies including NPC, such as linc00669 promotes nasopharyngeal carcinoma cell proliferation and invasion *via* competitively combining to the suppressor of cytokine signaling (SOCS1) (Qing et al., 2020). However, the functions of most lncRNAs in nasopharyngeal carcinoma remain elusive.

LncSPRY4 intronic transcript 1 (LncRNA SPRY4-IT1) is an intron of SPRY4 gene and the length of which is 708 bp. SPRY4-IT1 was discovered in melanoma cells as an oncogene for the first time (Khaitan et al., 2011). Increasing studies had demonstrated that SPRY4-IT1 was up-regulated in various cancers, such as the cervical cancer (Cao et al. 2016), the bladder cancer (Liu et al. 2017), and the esophageal cancer (Cui et al. 2016), and it was identified to promote tumor growth and metastasis. Up until now, the biological role and the potential molecular mechanisms of SPRY4-IT1 in nasopharyngeal carcinoma remains unknown. In this study, we identified the functions of SPRY4-IT1 on NPC cells *in vitro* and *in vivo*, which might shed new light on molecular therapeutics in NPC.

## Materials & Methods

### Cell culture

Human immortalized nasopharyngeal epithelial cell line (NP69) was obtained from Baiye Co, Ltd (Baiye, Shanghai, China) and cultured in keratinocyte/serum-free medium (Invitrogen, Grand Island, NY, USA). NPC cell lines (SUNE-1, 5-8F, CNE-1, HK1, 6-10B, CNE-2, HONE-1) were kindly provided by Professor Xia (Sun Yat-sen University Cancer Center, Guangzhou, China) and cultured in RPMI-1640 (Thermo Fisher Scientific, MA, USA) supplemented with 10% fetal bovine serum (FBS) (Thermo Fisher Scientific, MA, USA) and 100 U/mL penicillin and 100 µg/mL streptomycin (Thermo Fisher Scientific, MA, USA). All the cell lines were culture in a humidified incubator with 5% CO_2_ at 37 °C.

### Cell transfection

Small interfering RNA was employed to transiently transfect NPC cells, siRNA negative control (si-NC), siRNA against SPRY4-IT1 (si-SPRY4-IT1) were obtained from Gene Pharma (Shanghai, China), and the transfection of siRNA were conducted by using Lipo-fectamine3000 (Invitrogen, Carlsbad, CA, USA). Cells were seeded on six-well plates at a density of 5 × 10^4^ cells per well overnight. When cells reached 60% confluency, each well was transfected with 150 pmol siRNA and 3 µL Lipofectamine 3000. After 48 h of transfection, gene relative expression levels were detected by quantitative real-time PCR (qRT-PCR). The target sequences of the si-SPRY4-IT1 included: si-SPRY4-IT1-1 (CCCAGAATGTTGACAGCTGCCTCTT); si-SPRY4-IT1-2 (GCTTTCTGATTCCAAGGCCTATTAA). Lentivirus mediated-RNA interference technology was employed to establish stable SPRY4-IT1 knockdown cell line (HONE-1), the recombinant lentiviruses (LV-SPRY4-IT1 shRNA and LV-scrambled shRNA) were obtained from Genechem (Shanghai, China). Cells (5 × 10^4^) were seeded on six-well plates, the next day cells were infected with lentiviruses at a multiplicity of infection (MOI) of 50. After 72 h, cells were harvested for assessing gene expression level by qRT-PCR. The successfully infected cells were cultured in a selective medium containing 3 µg/mL puromycin (Invitrogen, Thermo Fisher Scientific) about 2–4 weeks to establish stably-infected cell line (HONE-1) for animal experiments. The target sequence of the Sh-SPRY4-IT1 (GCTTTCTGATTCCAAGG-CCTATTAA) was the same as the si-SPRY4-IT1-2 sequences.

### RNA extraction and qRT-PCR analysis

Total RNA was extracted from cultured cells with RNA-easy Isolation Reagent (Vazyme, China) and quantified by NanoDrop ND2000 (Thermo Fisher, Waltham, MA, USA). Extracted RNA was reverse-transcribed into complementary DNA (cDNA) by using a Reverse Transcription Kit (Accurate Biology, China). Real-time PCR analyses were performed with SYBR Green Pro Taq HS (Accurate Biology, China) and detected by AGS9600 System (bioanyu, China). The sequences of primers used were as follows: GAPDH, forward 5′-GACTCATGACCACAGTCCATGC-3′and reverse 5-AGAGGCAGGGATGATGTTCTG-3′; SPRY4-IT1, forward 5′- AGCCACATAAATTCAG-CAGA -3′and reverse 5′-CGATGTAGTAGGATTCCTTTCA-3′. The procedure included: step 1, 95 °C, 30 s; step 2, 95 °C, 5 s and 60 °C, 30 s, 45 amplification cycles; step 3, Dissociation stage. The expression of target genes was normalized according to the expression of GAPDH mRNA expression levels and were analyzed by the 2^−ΔΔ*Ct*^ method.

### Cell viability assay

Cell viability was examined by Cell Counting Kit-8 (CCK-8; Monmouth Junction, NJ, MedChemExpress, USA) and colony formation assay. After 48 h of transfection, transfected cells (2 × 10^3^ cells/well) were seeded into a 96-well plate and cultured at 37 °C, with 5% of CO_2_ for 6 h, 24 h, 48 h, 72 h, 96 h. The 10 µl CCK-8 reagent was added to each well and incubated at 37 °C for 2 h in dark, the absorbance at 450 nm was measured by a microplate reader (Molecular DevicesCMax Plus, USA). For colony formation assay, after 48 h of transfection, transfected cells (4 × 10^2^ cells/well) were seeded into 6-well plates for about 2 weeks and the medium was exchanged every other day. The cell colonies were fixed using 4% Paraformaldehyde (Bioss, Beijing, China) for 15 mins and stained using crystal violet (Sigma Life Science) for 30 mins at room temperature. The colony number was counted by manually.

### Transwell assays

The ability of cell migration was examined by the transwell assays. After 48 h of transfection, transfected cells (5 × 10^4^ cells/well) were seeded into the upper chamber (Corning Life Sciences, MA, USA) with 200 µL serum-free medium, and 600 µL10% serum-containing medium was added to the lower chamber. After incubation for 24 h, the cells on the upper membrane were wiped off by cotton swab, and the cells under membrane were fixed with 4% Paraformaldehyde (Bioss, Beijing, China) for 15mins, then stained with 0.1% crystal violet (Sigma Life Science) for 15mins and counted using inverted microscope in randomly selected fields.

### Wound healing assay

After 48 h of transfection, transfected cells (5 × 10^3^ cells/well) were seeded into 12-well plates. When cells reach 100% confluency, using a sterile 200 µL tip to make scratch and wash the cells thrice. Then cells were cultured in serum-free medium, the width of scratches was recorded by inverted microscope at 0 h, 24 h. Relative scratch healing ratio (%) = (scratch width after 24 h in each group/initial scratch width in each group)/(scratch width after 24 h in control group/initial scratch width in control group) ×100%.

### Flow cytometry

To evaluate the distribution of cell cycle phase, and the transfected cells were fixed with precooled 70% ethanol at −20 ^∘^C overnight. After cells were stained with the propidium iodide (PI) solution (BD Biosciences, Franklin Lakes, NJ, USA) for 15 mins, then measured by CytoFLEX flow cytometry (Beckman Coulter, Brea, CA, USA). The ModFit LT3.0 (VerifySoftware House, Topsham, ME, USA) was used for data analysis. In order to assess apoptotic rates, the transfected cells were collected. After cells were stained with FITC (BD Biosciences, Franklin Lakes, NJ, USA) and PI (BD Biosciences, Franklin Lakes, NJ, USA) for 15 mins, and detected by CytoFLEX flow cytometry (Beckman Coulter, Brea, CA, USA) and analyzed by FlowJo V10.0 (BD Biosciences, Franklin Lakes, NJ, USA).

### Western blotting assay

Total protein was extracted from cells using RIPA lysis buffer (P0013, Beyotime, Shanghai, China) with phosphatase inhibitors (HY-K0021; MedChemExpress, Shanghai, China) and protease inhibitors (ST506; Beyotime, Shanghai, China). Protein concentrations were quantified using the BCA protein assay kit (P0010; Beyotime, Shanghai, China). Protein was separated by SDS-polyacrylamide gel electrophoresis (SDS-PAGE) and then transferred to polyvinylidene difluoride membrane (ISEQ00010; Merck millipore, Darmstadt, Germany). Subsequently, the membrane was blocked with 5% nonfat milk at room temperature for 1 h and then incubated with primary antibodies at 4 °C overnight. Antibodies against *β*-actin (#8457, 1:1000, CST), Twist1 (#69366, 1:500, CST), Snail (#3879, 1:1,000, CST), cdc2 (#9116, 1:1000, CST), p-cdc2 (#4539, 1:1000, CST), cyclinB1 (#12231, 1:1000, CST), cleaved caspase-3 (#9664, 1:1000, CST), PARP (#9532, 1:1000, CST), Cleaved-PARP (#5625, 1:1000, CST), E-cadherin (ab231303, 1:1000, Abcam), Vimentin (ab8978, 1:1000, Abcam). Anti- *β*-actin used as the internal control. The second day, after being washed thrice with tris saline buffer with Tween (TBST), the membranes were incubated with the secondary antibodies (#7074 or #7076 1:5000, CST) at room temperature for 1 h. After being washed thrice with the TBST buffer, the protein bands were imaged with SuperSignal Western Blot Enhancer (#46,640; Thermo Fisher, Waltham, MA, USA) and the Tanon-5200 multi-imaging system (Tanon, Shanghai, China). ImageJ version 1.8.0 software (National Institutes of Health) was used to analyze the images.

### Immunofluorescence assay

Transfected cells were fixed in 4% paraformaldehyde for 20 mins and permeated with 0.3% Triton X-100 for 15 mins, then blocked with 5% bovine serum albumin (BSA) for 1 h at room temperature. The primary antibody E-cadherin (ab231303, 1:200, Abcam) and Vimentin (ab8978; 1:25, Abcam) was added and incubated overnight at 4 °C. The next day, Fluorophore-conjugated secondary antibodies # 4410 or # 4408, 1:2000; CST) were was added to the specimens and incubated for 1 h in the dark. DAPI was used to stain the nuclei in the dark. Finally, the slides were observed and images were captured under immunofluorescence microscope.

### Animal experiment

All athymic female mice (BLAB/c, aged 5 to 6 weeks) were obtained from Guangdong Medical Laboratory Animal Center (http://www.gdmlac.com.cn) and raised under the specific pathogen-free animal room according the national and international laws and policies. All mice were randomly divided into two groups (NC and Sh-SPRY4-IT1 groups), and received injections of cell suspension *via* the lateral tail vein at a density of 8 × 10^6^ cells/0.2 ml. All mice were sacrificed at 7 weeks after injection and the lungs were excised, fixed and stained with hematoxylin and eosin (H&E). For metastasis evaluation, the number of metastatic nodules was counted under microscopes as the previous study described (Fidler, 1986; Wang et al., 2014) (P250 Flash digital microscopes; 3DHISTECH, Budapest, Hungary).

### Ethical statement

All animal experiments were conducted by the institutional ethical guidelines of Sun Yat-Sen University and were approved by the ethical review committee from Animal Experimental Center of the Fifth Affiliated Hospital of Sun Yat-Sen University (approval no: 00165).

### Statistical analysis

All experiments were independently repeated three times, all the data was analyzed with GraphPad Prism 8.0 statistical software. The statistic difference between two groups was analyzed by unpaired two-tailed *t*-test or two-way analysis of variance (ANOVA) followed by Dunnett post-hoc test. The data are expressed as the means ± standard deviation (SD), and *P* < 0.05 was considered statistically significant.

## Results

### Expression of SPRY4-IT1 in NPC cell lines

**Figure 1:**
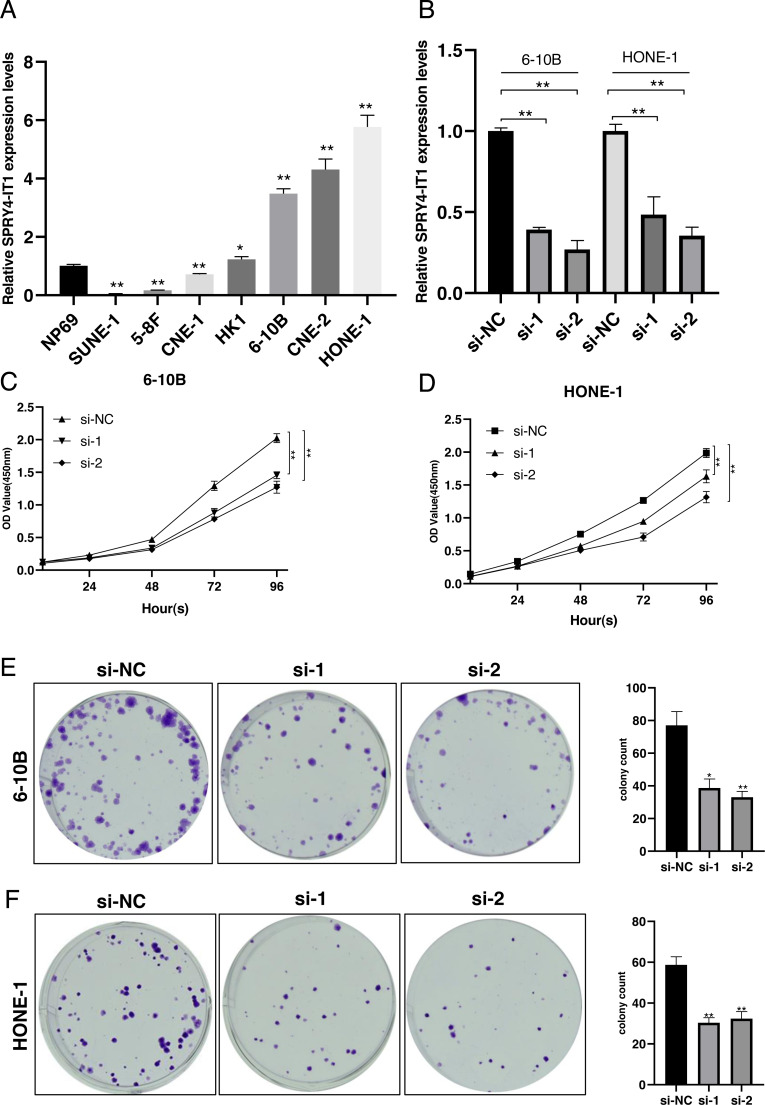
SPRY4-IT1 was up-regulated in several NPC cell lines and knockdown of SPRY4-IT1 inhibited cell proliferation. (A) The expression of SPRY4-IT1 was up-regulated in several NPC cell lines. We detected the expression of SPRY4-IT1 in NPC cell lines (SUNE-1, 5-8F, CNE-1, HK-1, 6-10B, CNE-2, HONE-1) and immortalized human normal nasopharyngeal epithelial cell line (NP69) through qRT-PCR. GAPDH was used as a standard control. (*n* = 3 for each group, unpaired two-tailed *t*-test, * *P* < 0.05, ** *P* < 0.01). (B) The expression level of SPRY4-IT1 in 6-10B and HONE-1 cells were measured by qRT- PCR, after SPRY4-IT knockdown, GAPDH was used as a standard control. (*n* = 3 for each group, unpaired two-tailed *t*-test, * *P* < 0.05, ** *P* < 0.01). (C–D) Four consecutive days of CCK-8 results showed that knockdown of SPRY4-IT1 suppressed cell proliferation. (*n* = 5 − 6 for each group, two-way ANOVA followed by Dunnett’s multiple comparisons test * *P* < 0.05, ** *P* < 0.01). (E–F) Colony formation results further confirmed that knockdown of SPRY4-IT1 suppressed cell proliferation. (*n* = 3 for each group, unpaired two-tailed *t*-test , * *P* < 0.05, ** *P* < 0.01).

We examined the expression of SPRY4-IT1 in seven nasopharyngeal carcinoma cell lines. The results showed that the expression level of SPRY4-IT1 was higher in HK1, 6-10B, HONE-1, and CNE-2 cell lines than the NP69 cell line, lower expression level of SPRY4-IT1 was identified in SUNE-1, 5-8F and CNE-1 cell lines compared with the NP69 cell line (Fig. 1A). The statistical results were presented in Table S1. The 6-10B and HONE-1 cell lines were selected for the subsequent experiments because the relatively higher expression level of SPRY4-IT1.

### Knockdown of SPRY4-IT1 inhibited proliferation of NPC cells

To investigate the effect of SPRY4-IT1 on cell proliferation, we silenced the SPRY4-IT1 expression of 6-10B and HONE-1 through transfection of si-SPRY4-IT1-1 and si-SPRY4-IT1-2. The transfection efficiency was detected by qRT-PCR (Fig. 1B). The statistical results were presented in Table S2. The CCK-8 results showed that cell proliferation was obviously inhibited in the si-SPRY4-IT1 group compared with the si-NC group (Figs. 1C and 1D). The statistical results were presented in Table S3. The Colony formation results showed that the number of cell colonies was decreased in the si-SPRY4-IT1 group compared with the si-NC group (Figs. 1E and 1F). The statistical results were presented in Table S4.

### Downregulation of SPRY4-IT1 induced cell cycle arrest and apoptosis

**Figure 2:**
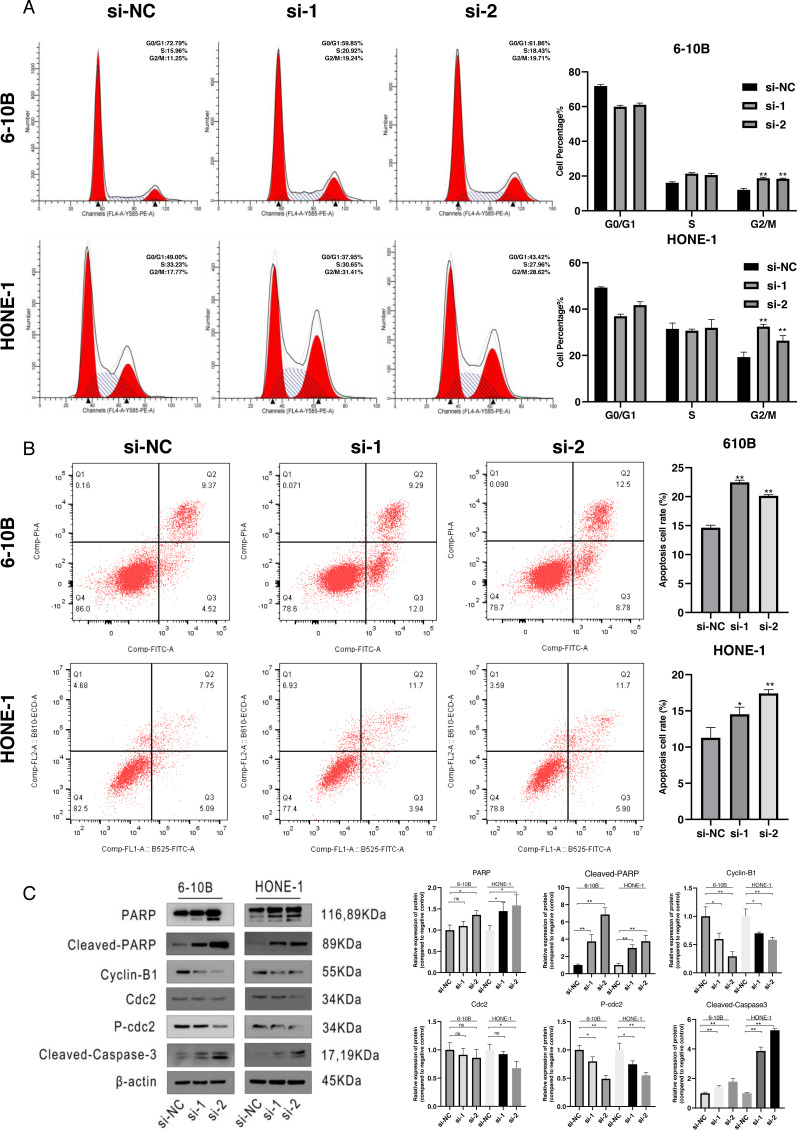
Cell cycle arrest and cell apoptosis were induced by silencing of SPRY4-IT1. (A) Transfected cells were harvested for flow cytometry. After knockdown of SPRY4-IT1, cell cycle percentage of G2 was significantly increased about 1.5−1.7 times. (*n* = 3 for each group, unpaired two-tailed *t*-test, *P < 0.05, **P < 0.01). (B) Cell apoptotic rate was significantly increased in the SPRY4-IT1 knockdown groups compare with the negative control group. (*n* = 3 − 4 for each group, unpaired two-tailed *t*-test, * *P* < 0.05, ** *P* < 0.01). (C) The expression of cell cycle related -proteins such as cyclin B1, cdc2 and p-cdc2 were down-regulated after knockdown of SPRY4-IT1; The expression of apoptotic biomarkers such as PARP, cleaved- PARP and cleaved-caspase-3 was up-regulated after knockdown of SPRY4-IT1. (*n* = 3 − 4 for each group, unpaired two-tailed *t*-test, * *P* < 0.05, ** *P* < 0.01).

To define the potential mechanisms of SPRY4-IT1 in the proliferation, we performed the flow cytometry. The results showed that SPRY4-IT1 knockdown led to cell cycle arrest at the G2/M phase and increased the cell apoptosis rate (Figs. 2A and 2B). The statistical results were presented in Tables S5 and S6. Consistently, the Western blotting results showed that the expression of cell cycle-related proteins (cyclin B1, cdc2 and p-cdc2) were down-regulated, and several apoptotic biomarkers (PARP, cleaved-PARP, cleaved-caspase-3) were up-regulated (Fig. 2C). The statistical results were presented in Tables S7 and S8.

### Knockdown of SPRY4-IT1 suppressed the NPC cell migration *in vitro* and metastasis *in vivo*

**Figure 3:**
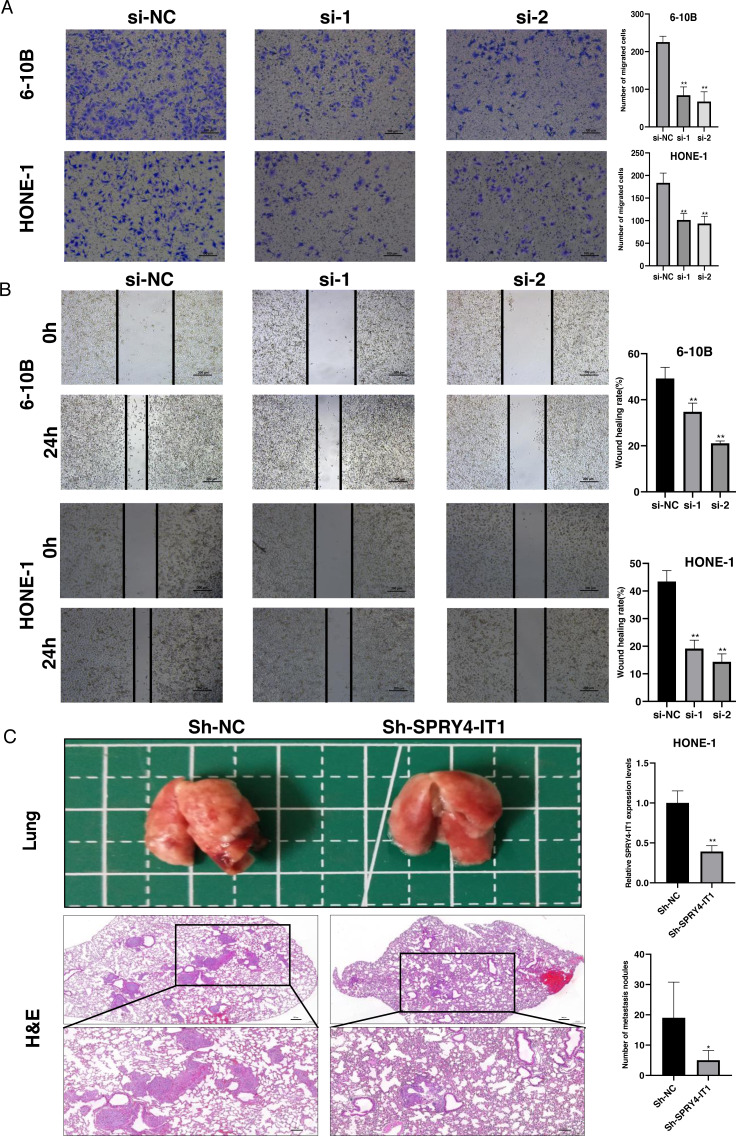
Knockdown of SPRY4-IT1 inhibited the NPC cell migration *in vitro* and metastasis *in vivo*. (A–B) The migration of 6-10B and HONE-1 cells were assessed by transwell assays (scale bar, 100 µm) and wound healing assay (scale bar, 200 µm) after knockdown of SPRY4-IT1. (C) Images of the representative lung and representative HE staining of lung metastatic tumors. (*n* = 5 − 6 for each group, unpaired two-tailed *t*-test, * *P* < 0.05, ** *P* < 0.01).

The transwell assay and the wound healing assay were used to observe the effect of SPRY4-IT1 on cell migration. The results showed that the cell mobility of the si-SPRY4-IT1 group was decreased compared with the si-NC group (Fig. 3A). The statistical results were presented in Table S9. Consistently, the scratch width of the si-SPRY4-IT1 group was wider than that of si-NC group (Fig. 3B). The statistical results were presented in Table S10. To further determine the effect of SPRY4-IT1 on metastasis *in vivo*, we performed the metastatic model of BALB/c nude mice by tail vein injection of HONE-1- Sh-SPRY4-IT1 cells or HONE-1-Sh-NC cells, respectively. The results showed that knockdown of SPRY4-IT1 decreased the number of lung metastatic nodules compared with the negative control group, consistent with the vitro results (Fig. 3C). The statistical results were presented in Tables S11 and S12.

### Knockdown of SPRY4-IT1 affected EMT-related proteins expression in NPC cells

**Figure 4:**
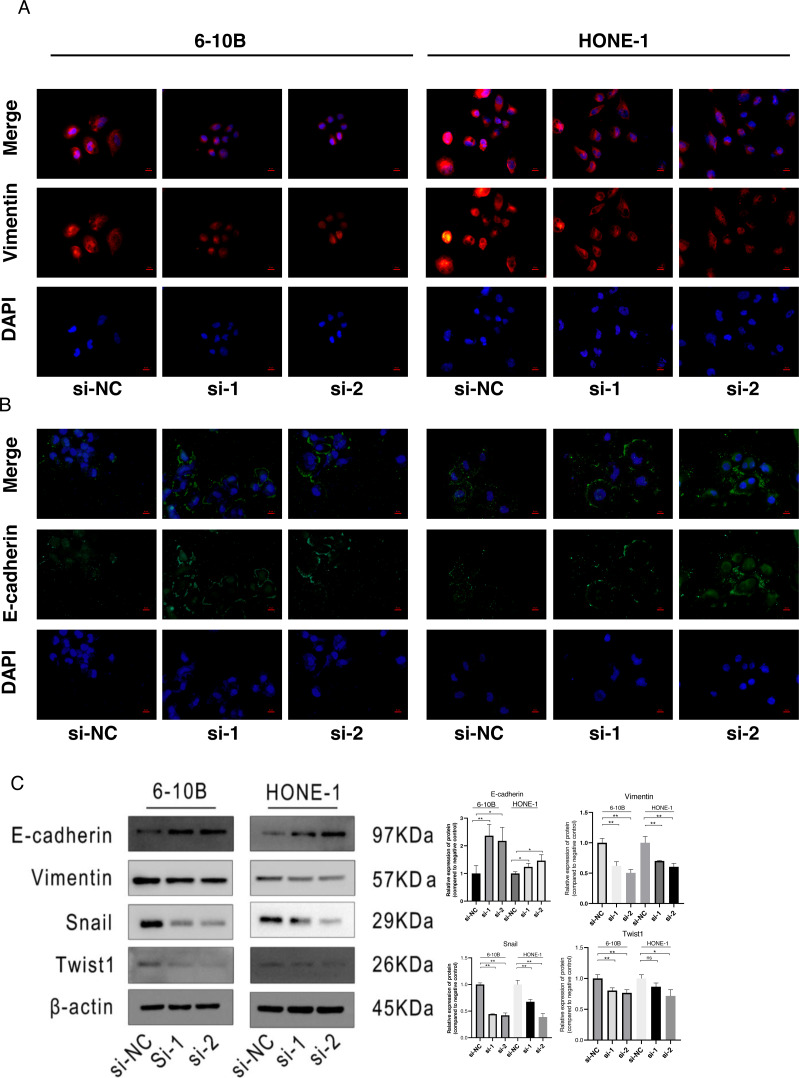
Knockdown of SPRY4-IT1 affected EMT-related proteins expression. (A) Immunofluorescence of Vimentin (red), E-cadherin (green) and nuclei stained with DAPI (blue) in 6-10B and HONE-1 cell lines (scale bar, 20 µm). (B) Quantification of western blotting analysis showed EMT-related proteins expression in 6-10B and HONE-1 cell lines, the expression of E-cadherin was up-regulated and the expression of Vimentin, Snail and Twist1 were down-regulated. (*n* = 3, for each group, unpaired two-tailed *t*-test, * *P* < 0.05, ** *P* < 0.01).

The immunofluorescence was performed to detect the expression changes of E-cadherin and Vimentin after SPRY4-IT1 knockdown. The images showed that the expression of E-cadherin displayed ascending trend, while the expression of Vimentin exhibited descending trend in the si-SPRY4-IT1 group compared with the si-NC group (Figs. 4A and 4B). Consistently, the Western blotting results showed that the expression of E-cadherin was up-regulated, and the expression of Vimentin, Snail, and Twist1 were down-regulated (4B). The statistical results were presented in Table S13.

## Discussion

Nasopharyngeal carcinoma is a distinct head and neck squamous cell carcinoma with a high incidence rate in Southeast Asia, Northern Africa and Greenland, and the metastasis and recurrence are main causes of poor prognosis (Chang et al., 2021). With the development of lncRNAs analysis, the aberrant expressions of certain lncRNAs tightly related to the progression of NPC. Identification of new tumor-specific lncRNAs might provide a potential target for the therapy (Tang & He, 2021). SPRY4-IT1 was derived from an intron of the SPRY4 gene and largely localized in the cytoplasm, which originally identified in melanoma (Khaitan et al., 2011). We revealed that the expression level of SPRY4-IT1 in NPC cell lines compared with NP69 cell line for the first time. Moreover, knockdown of SPRY4-IT1 inhibited the cell proliferation, migration, metastasis, and promoted cell cycle arrest and apoptosis.

Cell cycle is a complex process that regulated by numerous molecular activities and proteins expression levels. Cyclin-dependent kinase 1 (CDK1), also considered as cell division control protein 2 (cdc2), was the major regulator for the cell switch from the G2 phase to mitosis (Prevo et al., 2018). Cyclin B1 was regarded as the primary partner of CDK1, and the cyclin B1-CDK1 complex served as a key role in mitotic entry (Li, Qian & Sun, 2019). In eukaryotic cell mitosis, cyclin B1 transfer to the nucleus during prophase, and high expression levels of cyclin B1 leading to the formation of cyclin B1-CDK1 complex during the late G2 phase (Wijnen et al., 2021). The activity of the cyclin B1-CDK1 complex was suppressed by inhibitory phosphorylation sites of threonine 14 and tyrosine 15 of the CDK1, when the inhibitory phosphates were removed by cdc25, the cyclin B1-CDK1 complex was activated and then the cells entry into mitosis (Prevo et al., 2018). Our study demonstrated that knockdown of SPRY4-IT1 decreased the expression levels of cyclin B1, cdc2 and p-cdc2, along with the failure of formation and activation of the cyclin B1-CDK1 complex, led to cell cycle arrest at the G2/M phase. When the cell cycle arrested at checkpoint, and failed to repair DNA damage would result in cell death (Lin et al., 2021; Liu et al., 2006). Apoptosis is a common type of cell death and induced by the activation of the apoptotic caspases-9, intrinsically and extrinsically (Hotchkiss et al., 2009). The fundamental caspase-dependent pathways included death receptor-mediated caspase-8 and the mitochondria-dependent caspase-9 pathways. In apoptosis process, the activation of caspase-9 and caspase-8 induced the activation of caspase-3, and PARP acted as downstream substrate of caspase-3, leading to cell apoptosis (Tower, 2015). Our study demonstrated that the expression levels of PARP, cleaved-PARP and cleaved-caspase-3 were up-regulated after knockdown of SPRY4-IT1, which indicated that knockdown of SPTY4-IT1 induced apoptosis might through the mitochondria-dependent caspase-3 pathway.

Metastasis closely associate with the cancer deaths, and regulated by multiple genes through the acquisition of molecular and phenotypic changes result in cancer cells transferring from a primary location to distant organ (Liu et al., 2021). The epithelial to mesenchymal transition (EMT) usually considered as the key processes of tumor metastasis. During EMT, the cells gradually lose features of epithelial cell and acquire characteristics of mesenchymal cells, this process involved the activation of EMT-inducing transcription factors (EMT-TFs), and subsequently accompanied with the decrease of epithelial markers expression and the increase of mesenchymal markers expression (Bakir et al., 2020). E-cadherin is a typical epithelial marker and it is regarded as the master regulator of cell adhesion and repressed by Snail family. On the contrary, Vimentin is a canonical mesenchymal marker and it is considered as the active player of cell migration and activated by Twist family (Jayachandran, Srinivasan & Mani, 2021). In our study, knockdown of SPRY4-IT1 decreased the protein expression levels of Snail, Twist1 and Vimentin, and increased the protein expression level of E-cadherin, which suggested that knockdown of SPRY4-IT1 inhibited NPC cell metastasis might *via* the regulation of EMT pathway.

## Conclusions

In summary, the present study demonstrated that SPRY4-IT1 expression was dysregulated in NPC cell lines. Moreover, knockdown of SPRY4-IT1 inhibited cell proliferation, migration and metastasis in NPC. These preliminary findings indicated that SPRY4-IT1 plays a key role in the progression of NPC. However, this study has some limitations, the underlying molecular mechanisms between SPRY4-IT1 and EMT remain unknown. Therefore, further studies are needed to be conducted.

##  Supplemental Information

10.7717/peerj.13221/supp-1 Supplemental Information 1 Statistical analysis of the expression levels of SPRY4-IT1 in NPC cells

10.7717/peerj.13221/supp-2 Supplemental Information 2 Statistical analysis of the expression level of SPRY4-IT1 knockdown

10.7717/peerj.13221/supp-3 Supplemental Information 3 Statistical analysis of CCK-8 results

10.7717/peerj.13221/supp-4 Supplemental Information 4 Statistical analysis of cell colony count

10.7717/peerj.13221/supp-5 Supplemental Information 5 Statistical analysis of percentage of G2/M

10.7717/peerj.13221/supp-6 Supplemental Information 6 Statistical analysis of cell apoptosis rate

10.7717/peerj.13221/supp-7 Supplemental Information 7 Statistical analysis of cell cycle-related proteins expression

10.7717/peerj.13221/supp-8 Supplemental Information 8 Statistical analysis of apoptosis-related proteins expression

10.7717/peerj.13221/supp-9 Supplemental Information 9 Statistical analysis of transwell assay results

10.7717/peerj.13221/supp-10 Supplemental Information 10 Statistical analysis of the wound healing rate

10.7717/peerj.13221/supp-11 Supplemental Information 11 Statistical analysis of the expression level of SPRY4-IT1 stable knockdown

10.7717/peerj.13221/supp-12 Supplemental Information 12 Statistical analysis of the number of the lung metastatic nodules

10.7717/peerj.13221/supp-13 Supplemental Information 13 Statistical analysis of EMT-related proteins expression

## Additional Information and Declarations

### Data Availability

The following information was supplied regarding data availability: The data is available at Figshare: Li, yanfei (2022): Long Non-Coding RNA SPRY4-IT1 promotes proliferation and metastasis in nasopharyngeal carcinoma cell. figshare. Dataset. https://doi.org/10.6084/m9.figshare.18696659.v2.
