# Supplementary material for: Long non-coding RNA SPRY4-IT1 promotes proliferation and metastasis in nasopharyngeal carcinoma cell
Source: PeerJ. 2022 Mar 30;10:e13221. doi: 10.7717/peerj.13221 (PMC8976472; doi:10.7717/peerj.13221)
Supplement: Supplemental Information 1 [file peerj-10-13221-s001.docx]

| **Cells** | **2^−ΔΔCt^ (mean ± SD)** | ***p*-value** | **df** |
| --- | --- | --- | --- |
| NP69 | 1.011 ± 0.04453 | - | - |
| SUNE-1 | 0.03440 ± 0.02258 | **< 0.001** | 4 |
| 5-8F | 0.1684 ± 0.005374 | **< 0.001** | 4 |
| CNE-1 | 0.7165 ± 0.02651 | **< 0.001** | 4 |
| HK1 | 1.231 ± 0.09194 | **0.02** | 4 |
| 6-10B | 3.483 ± 0.1668 | **< 0.001** | 4 |
| CNE-2 | 4.307 ± 0.3637 | **< 0.001** | 4 |
| HONE-1 | 5.772 ± 0.3963 | **< 0.001** | 4 |

**Table S1 Statistical analysis of the expression levels of SPRY4-IT1 in NPC cells**

**Notes.**

Significantly different for p-values < 0.05 indicated in bold.
