# Supplementary material for: Long non-coding RNA SPRY4-IT1 promotes proliferation and metastasis in nasopharyngeal carcinoma cell
Source: PeerJ. 2022 Mar 30;10:e13221. doi: 10.7717/peerj.13221 (PMC8976472; doi:10.7717/peerj.13221)
Supplement: Supplemental Information 2 [file peerj-10-13221-s002.docx]

**Table S2 Statistical analysis of the expression level of SPRY4-IT1 knockdown**

| **Group** | **2^-ΔΔct^ (mean ± SD)** | ***p*-value** | **df** |
| --- | --- | --- | --- |
| 6-10B-si-NC | 1.000 ± 0.09815 | - | - |
| 6-10B-si-1 | 0.4589 ± 0.1155 | **0.0035** | 4 |
| 6-10b-si-2 | 0.2417 ± 0.06200 | **< 0.001** | 4 |
| HONE-1-si-NC | 1.000 ± 0.04163 | **-** | - |
| HONE-1-si-1 | 0.4145 ± 0.1436 | **0.0025** | 4 |
| HONE-1-si-2 | 0.3544 ± 0.05200 | **< 0.001** | 4 |

**Notes.**

Significantly different for p-values < 0.05 indicated in bold.
