# Supplementary material for: Long non-coding RNA SPRY4-IT1 promotes proliferation and metastasis in nasopharyngeal carcinoma cell
Source: PeerJ. 2022 Mar 30;10:e13221. doi: 10.7717/peerj.13221 (PMC8976472; doi:10.7717/peerj.13221)
Supplement: Supplemental Information 3 [file peerj-10-13221-s003.docx]

**Table S3 Statistical analysis of CCK-8 results**

| **Group** | | **OD values (mean ± SD)** | ***p*-value** | **df** |
| --- | --- | --- | --- | --- |
| 24 hours | 6-10B-si-NC | 0.2371 ± 0.008572 | - | - |
|  | 6-10B-si-1 | 0.2371 ± 0.008572 | **0.0006** | 7.861 |
|  | 6-10b-si-2 | 0.1767 ± 0.008124 | **<0.0001** | 7.512 |
|  | HONE-1-si-NC | 0.3395 ± 0.01439 | **-** | - |
|  | HONE-1-si-1 | 0.2655 ± 0.01276 | **0.0055** | 6.591 |
|  | HONE-1-si-2 | 0.2598 ± 0.02160 | **0.0042** | 8.955 |
| 48 hours | 6-10B-si-NC | 0.4702 ± 0.01819 | **-** | - |
|  | 6-10B-si-1 | 0.3381 ± 0.02878 | **0.0003** | 6.054 |
|  | 6-10b-si-2 | 0.3132 ± 0.01412 | **<0.0001** | 7.925 |
|  | HONE-1-si-NC | 0.7522 ± 0.03647 | **-** | - |
|  | HONE-1-si-1 | 0.5717 ± 0.02434 | **0.0051** | 5.945 |
|  | HONE-1-si-2 | 0.5055 ± 0.03292 | **0.0007** | 6.198 |
| 72 hours | 6-10B-si-NC | 1.294 ± 0.07262 | **-** | - |
|  | 6-10B-si-1 | 0.8802 ± 0.06429 | **0.0001** | 6.562 |
|  | 6-10b-si-2 | 0.7822 ± 0.01932 | **0.0002** | 4.542 |
|  | HONE-1-si-NC | 1.265 ± 0.02835 | **-** | - |
|  | HONE-1-si-1 | 0.9474 ± 0.02759 | **0.0006** | 9.364 |
|  | HONE-1-si-2 | 0.7102 ± 0.06090 | **<0.0001** | 8.351 |
| 96 hours | 6-10B-si-NC | 2.042 ± 0.05871 | **-** | - |
|  | 6-10B-si-1 | 1.454 ± 0.05288 | **<0.0001** | 7.644 |
|  | 6-10b-si-2 | 1.254 ± 0.07867 | **<0.0001** | 7.227 |
|  | HONE-1-si-NC | 1.987 ± 0.06990 | **-** | - |
|  | HONE-1-si-1 | 1.631 ± 0.09938 | **0.0017** | 109.992 |
|  | HONE-1-si-2 | 1.316 ±0.08565 | **<0.0001** | 8.412 |

**Notes.**

Significantly different for p-values < 0.05 indicated in bold.

two-way analysis of variance (ANOVA) followed by Dunnett post-hoc test
