# Supplementary material for: Long non-coding RNA SPRY4-IT1 promotes proliferation and metastasis in nasopharyngeal carcinoma cell
Source: PeerJ. 2022 Mar 30;10:e13221. doi: 10.7717/peerj.13221 (PMC8976472; doi:10.7717/peerj.13221)
Supplement: Supplemental Information 4 [file peerj-10-13221-s004.docx]

**Table S4 Statistical analysis of cell colony count**

| **Group** | **Colony count (mean ± SD)** | ***p*-value** | **df** |
| --- | --- | --- | --- |
| 6-10B-si-NC | 77.00 ± 8.485 | - | - |
| 6-10B-si-1 | 38.67 ± 5.508 | **0.0132** | 4 |
| 6-10b-si-2 | 33.00 ± 3.606 | **0.0063** | 4 |
| HONE-1-si-NC | 58.67 ± 4.041 | **-** | - |
| HONE-1-si-1 | 30.33 ± 2.517 | **0.0006** | 4 |
| HONE-1-si-2 | 32.33 ± 3.512 | **0.0012** | 4 |

**Notes.**

Significantly different for p-values < 0.05 indicated in bold.
