# Supplementary material for: Long non-coding RNA SPRY4-IT1 promotes proliferation and metastasis in nasopharyngeal carcinoma cell
Source: PeerJ. 2022 Mar 30;10:e13221. doi: 10.7717/peerj.13221 (PMC8976472; doi:10.7717/peerj.13221)
Supplement: Supplemental Information 5 [file peerj-10-13221-s005.docx]

| **Group** | **percentage of G2/M phase (mean ± SD)** | ***p*-value** | **df** |
| --- | --- | --- | --- |
| 6-10B-si-NC | 12.05 ±0.8481 | **-** | - |
| 6-10B-si-1 | 18.71 ±0.5856 | **0.0004** | 4 |
| 6-10b-si-2 | 18.43 ±0.3550 | **0.0003** | 4 |
| HONE-1-si-NC | 19.28 ±2.135 | **-** | - |
| HONE-1-si-1 | 32.44 ±1.051 | **0.0002** | 4 |
| HONE-1-si-2 | 26.36 ±2.235 | **0.0099** | 4 |

**Table S5 Statistical analysis of percentage of G2/M**

**Notes.**

Significantly different for p-values < 0.05 indicated in bold.
