# Supplementary material for: Long non-coding RNA SPRY4-IT1 promotes proliferation and metastasis in nasopharyngeal carcinoma cell
Source: PeerJ. 2022 Mar 30;10:e13221. doi: 10.7717/peerj.13221 (PMC8976472; doi:10.7717/peerj.13221)
Supplement: Supplemental Information 6 [file peerj-10-13221-s006.docx]

**Table S6 Statistical analysis of cell apoptosis rate**

| **Group** | **percentage of cell apoptosis (mean ± SD)** | ***p*-value** | **df** |
| --- | --- | --- | --- |
| 6-10B-si-NC | 14.64 ± 0.4270 | - | - |
| 6-10B-si-1 | 22.48 ± 0.3686 | **<0.0001** | 6 |
| 6-10b-si-2 | 20.13 ± 0.2235 | **<0.0001** | 6 |
| HONE-1-si-NC | 11.27 ± 1.440 | - | - |
| HONE-1-si-1 | 14.52 ± 0.9851 | **0.0322** | 4 |
| HONE-1-si-2 | 17.40 ± 0.5292 | **0.0023** | 4 |

**Notes.**

Significantly different for p-values < 0.05 indicated in bold.
