# Supplementary material for: Long non-coding RNA SPRY4-IT1 promotes proliferation and metastasis in nasopharyngeal carcinoma cell
Source: PeerJ. 2022 Mar 30;10:e13221. doi: 10.7717/peerj.13221 (PMC8976472; doi:10.7717/peerj.13221)
Supplement: Supplemental Information 7 [file peerj-10-13221-s007.docx]

| **Group** | | **Relative gray value values (mean ± SD)** | ***p*-value** | **df** |
| --- | --- | --- | --- | --- |
| Cdc2 | 6-10B-si-NC | 1.000 ± 0.09890 | - | - |
|  | 6-10B-si-1 | 0.6910 ± 0.09619 | 0.4274 | 4 |
|  | 6-10b-si-2 | 0.4967 ± 0.05863 | 0.2843 | 4 |
|  | HONE-1-si-NC | 1.000 ± 0.1003 | - | - |
|  | HONE-1-si-1 | 0.9245 ± 0.04983 | 0.3074 | 4 |
|  | HONE-1-si-2 | 0.6762 ± 0.1221 | **0.0238** | 4 |
| P-cdc2 | 6-10B-si-NC | 1.000 ± 0.08327 | - | - |
|  | 6-10B-si-1 | 0.7971 ± 0.08142 | 0.0392 | 4 |
|  | 6-10b-si-2 | 0.4889 ± 0.06248 | **0.001** | 4 |
|  | HONE-1-si-NC | 1.000 ± 0.1211 | - | - |
|  | HONE-1-si-1 | 0.7474 ± 0.05982 | **0.0317** | 4 |
|  | HONE-1-si-2 | 0.5503 ± 0.05007 | **0.0040** | 4 |
| CyclinB1 | 6-10B-si-NC | 1.000 ± 0.1671 | - | - |
|  | 6-10B-si-1 | 0.5980 ± 0.1054 | **0.0244** | 4 |
|  | 6-10b-si-2 | 0.2915 ± 0.08197 | **0.0027** | 4 |
|  | HONE-1-si-NC | 1.000 ± 0.1300 | - | - |
|  | HONE-1-si-1 | 0.6989 ± 0.02402 | **0.0169** | 4 |
|  | HONE-1-si-2 | 0.5852 ± 0.04392 | **0.0064** | 4 |

**Table S7 Statistical analysis of cell cycle-related proteins expression**

**Notes.**

Significantly different for p-values < 0.05 indicated in bold.
