# Supplementary material for: Long non-coding RNA SPRY4-IT1 promotes proliferation and metastasis in nasopharyngeal carcinoma cell
Source: PeerJ. 2022 Mar 30;10:e13221. doi: 10.7717/peerj.13221 (PMC8976472; doi:10.7717/peerj.13221)
Supplement: Supplemental Information 8 [file peerj-10-13221-s008.docx]

| **Group** | | **Relative**  **gray values**  **(mean ± SD)** | ***p*-value** | **df** |
| --- | --- | --- | --- | --- |
| PARP | 6-10B-si-NC | 1.000 ± 0.1211 | - | - |
|  | 6-10B-si-1 | 1.098 ± 0.1050 | 0.3492 | 4 |
|  | 6-10b-si-2 | 1.358 ± 0.1008 | **0.0170** | 4 |
|  | HONE-1-si-NC | 1.000 ± 0.1083 | - | - |
|  | HONE-1-si-1 | 1.447 ± 0.1975 | **0.0264** | 4 |
|  | HONE-1-si-2 | 1.580 ± 0.2507 | **0.0212** | 4 |
|  | 6-10B-si-NC | 1.000 ± 0.1240 | **-** | - |
|  | 6-10B-si-1 | 3.739 ± 0.8065 | **0.0044** | 4 |
|  | 6-10b-si-2 | 6.864 ± 0.7842 | **0.0002** | 4 |
| Cleaved-PARP | HONE-1-si-NC | 1.000 ± 0.2170 | **-** | - |
|  | HONE-1-si-1 | 2.991 ± 0.3679 | **0.0013** | 4 |
|  | HONE-1-si-2 | 3.760 ± 0.6837 | **0.0026** | 4 |
| Cleaved-caspase-3 | 6-10B-si-NC | 1.000 ± 0.07768 | - | - |
|  | 6-10B-si-1 | 1.407 ± 0.1138 | **0.0069** | 4 |
|  | 6-10b-si-2 | 1.786 ± 0.2136 | **0.0039** | 4 |
|  | HONE-1-si-NC | 1.000 ± 0.05867 | - | - |
|  | HONE-1-si-1 | 3.864 ± 0.2663 | **<0.0001** | 4 |
|  | HONE-1-si-2 | 5.261 ± 0.1141 | **<0.0001** | 4 |

**Table S8 Statistical analysis of apoptosis-related proteins expression**

**Notes.**

Significantly different for p-values < 0.05 indicated in bold.
