# Supplementary material for: Long non-coding RNA SPRY4-IT1 promotes proliferation and metastasis in nasopharyngeal carcinoma cell
Source: PeerJ. 2022 Mar 30;10:e13221. doi: 10.7717/peerj.13221 (PMC8976472; doi:10.7717/peerj.13221)
Supplement: Supplemental Information 9 [file peerj-10-13221-s009.docx]

**Table S9 Statistical analysis of transwell assay results**

| **Group** | **migrated cells count (mean ± SD)** | ***p*-value** | **df** |
| --- | --- | --- | --- |
| 6-10B-si-NC | 225.3 ± 15.56 | - | - |
| 6-10B-si-1 | 84.00 ± 22.44 | **<0.0001** | 6 |
| 6-10b-si-2 | 67.25 ± 26.00 | **<0.0001** | 6 |
| HONE-1-si-NC | 183.7 ± 21.73 | - | - |
| HONE-1-si-1 | 101.5 ± 14.55 | **0.0018** | 5 |
| HONE-1-si-2 | 93.67 ± 16.07 | **0.0045** | 5 |

**Notes.**

Significantly different for p-values < 0.05 indicated in bold.
