# Supplementary material for: Long non-coding RNA SPRY4-IT1 promotes proliferation and metastasis in nasopharyngeal carcinoma cell
Source: PeerJ. 2022 Mar 30;10:e13221. doi: 10.7717/peerj.13221 (PMC8976472; doi:10.7717/peerj.13221)
Supplement: Supplemental Information 10 [file peerj-10-13221-s010.docx]

**Table S10 Statistical analysis of the wound healing rate**

| **Group** | **wound healing rate (mean ± SD)** | ***p*-value** | **df** |
| --- | --- | --- | --- |
| 6-10B-si-NC | 49.21 ± 4.863 | - | - |
| 6-10B-si-1 | 34.76 ± 3.764 | **0.0033** | 6 |
| 6-10b-si-2 | 21.06 ± 1.025 | **<0.0001** | 6 |
| HONE-1-si-NC | 43.44 ± 3.952 | **-** | - |
| HONE-1-si-1 | 19.14 ± 3.062 | **0.0018** | 6 |
| HONE-1-si-2 | 14.36 ± 2.882 | **0.0045** | 9 |

**Notes.**

Significantly different for p-values < 0.05 indicated in bold.
