# Supplementary material for: Long non-coding RNA SPRY4-IT1 promotes proliferation and metastasis in nasopharyngeal carcinoma cell
Source: PeerJ. 2022 Mar 30;10:e13221. doi: 10.7717/peerj.13221 (PMC8976472; doi:10.7717/peerj.13221)
Supplement: Supplemental Information 11 [file peerj-10-13221-s011.docx]

**Table S11 Statistical analysis of the expression level of SPRY4-IT1 stable knockdown**

| **Group** | **2^-ΔΔct^ (mean ± SD)** | ***p*-value** | **df** |
| --- | --- | --- | --- |
| HONE-1-Sh-NC | 1.000 ± 0.1519 | **-** | - |
| HONE-1-Sh-SPRY4-IT1 | 0.3923 ± 0.07345 | **0.0034** | 4 |

**Notes.**

Significantly different for p-values < 0.05 indicated in bold.
