# Supplementary material for: Long non-coding RNA SPRY4-IT1 promotes proliferation and metastasis in nasopharyngeal carcinoma cell
Source: PeerJ. 2022 Mar 30;10:e13221. doi: 10.7717/peerj.13221 (PMC8976472; doi:10.7717/peerj.13221)
Supplement: Supplemental Information 12 [file peerj-10-13221-s012.docx]

**Table S12 Statistical analysis of the number of the lung metastatic nodules**

| **Group** | **2^-ΔΔct^ (mean ± SD)** | ***p*-value** | **df** |
| --- | --- | --- | --- |
| HONE-1-Sh-NC | 19.00 ± 11.78 | **-** | - |
| HONE-1-Sh-SPRY4-IT1 | 5.000± 3.225 | **0.0034** | 10 |

**Notes.**

Significantly different for p-values < 0.05 indicated in bold.
