# Supplementary material for: Long non-coding RNA SPRY4-IT1 promotes proliferation and metastasis in nasopharyngeal carcinoma cell
Source: PeerJ. 2022 Mar 30;10:e13221. doi: 10.7717/peerj.13221 (PMC8976472; doi:10.7717/peerj.13221)
Supplement: Supplemental Information 13 [file peerj-10-13221-s013.docx]

**Table S13 Statistical analysis of EMT-related proteins expression**

| **Group** | | **Relative gray value values (mean ± SD)** | ***p*-value** | **df** |
| --- | --- | --- | --- | --- |
| E-cadherin | 6-10B-si-NC  6-10B-si-1  6-10b-si-2  HONE-1-si-NC  HONE-1-si-1  HONE-1-si-2 | 1.000 ± 0.2831  2.367 ± 0.4064  2.179 ± 0.4858  1.000 ± 0.06310  1.238 ± 0.1260  1.463 ± 0.2146 | -  **0.0088**  **0.0221**  -  **0.0431**  **0.0230** | -  4  4  -  4  4 |
| β-catenin | 6-10B-si-NC | 1.000 ± 0.1994 | - | - |
|  | 6-10B-si-1 | 2.085 ± 0.2380 | **0.0038** | 4 |
|  | 6-10b-si-2 | 2.056 ± 0.2564 | **0.0049** | 4 |
|  | HONE-1-si-NC | 1.000 ± 0.08865 | - | - |
|  | HONE-1-si-1 | 1.526 ± 0.09469 | **0.0022** | 4 |
|  | HONE-1-si-2 | 1.552 ± 0.1102 | **0.0025** | 4 |
| Vimentin | 6-10B-si-NC | 1.000 ± 0.07226 | - | - |
|  | 6-10B-si-1 | 0.6208 ± 0.06690 | **0.0026** | 4 |
|  | 6-10b-si-2 | 0.5047 ± 0.05492 | **< 0.001** | 4 |
|  | HONE-1-si-NC | 1.000 ± 0.1031 | - | - |
|  | HONE-1-si-1 | 0.7012 ± 0.007405 | **0.0369** | 4 |
|  | HONE-1-si-2 | 0.6039 ± 0.05575 | **0.0042** | 4 |
| Snail | 6-10B-si-NC | 1.000 ± 0.03036 | - | - |
|  | 6-10B-si-1 | 0.4443 ± 0.005011 | **< 0.001** | 4 |
|  | 6-10b-si-2 | 0.4201 ± 0.04545 | **< 0.001** | 4 |
|  | HONE-1-si-NC | 1.000 ± 0.07764 | - | - |
|  | HONE-1-si-1 | 0.6752 ± 0.04564 | **0.0033;** | 4 |
|  | HONE-1-si-2 | 0.3895 ± 0.06667 | **< 0.001** | 4 |
| Twist1 | 6-10B-si-NC | 1.000 ± 0.06207 | - | - |
|  | 6-10B-si-1 | 0.8046 ± 0.04310 | **0.0110** | 4 |
|  | 6-10b-si-2 | 0.7670 ± 0.05010 | **0.0072** | 4 |
|  | HONE-1-si-NC | 1.000 ± 0.06097 | - | - |
|  | HONE-1-si-1 | 0.8651 ± 0.06218 | 0.0550 | 4 |
|  | HONE-1-si-2 | 0.7165 ± 0.1008 | **0.0140** | 4 |

**Notes.**

Significantly different for p-values < 0.05 indicated in bold.
